# User satisfaction with the National Health Insurance Program: A community-based survey from the Ilam district of Nepal

**DOI:** 10.1371/journal.pone.0303045

**Published:** 2024-05-24

**Authors:** Rasmita Shrestha, Aditya Shakya, Pratik Khanal, Vijay Kumar Khanal, Nilambar Jha, Gyanu Nepal Gurung, Laxmi Subedi

**Affiliations:** 1 School of Public Health and Community Medicine, B.P. Koirala Institute of Health Sciences, Dharan, Nepal; 2 Health Office Ilam, Ministry of Health, Koshi Province, Nepal; 3 Nepal Public Health Association, Lalitpur, Nepal; 4 Bergen Center for Ethics and Priority Setting in Health (BCEPS), Department of Global Public Health and Primary Care, University of Bergen, Bergen, Norway; University of Bergen: Universitetet i Bergen, NORWAY

## Abstract

**Background:**

The Government of Nepal initiated a family-based National Health Insurance Program (NHIP) in April 2016, aiming to ensure universal health coverage (UHC) by enhancing access to and utilization of quality health services. However, NHIP, in its initial years of implementation, encountered challenges such as low population coverage, a high dropout rate, and concerns among the insured regarding the quality of healthcare services. There is a dearth of information regarding user satisfaction with the NHIP in Nepal. This study aimed to assess user satisfaction with NHIP at the household level in Nepal.

**Methods:**

We conducted a cross-sectional study among 347 households in the Ilam district using a multi-stage random sampling method. Face-to-face interviews were conducted with household heads enrolled in NHIP. A semi-structured questionnaire was used to collect the data. The multivariable logistic regression analysis was done to identify the predictors of satisfaction level.

**Results:**

Overall, 53.6% of the insured were satisfied with the NHIP, while 31.1% had comprehensive knowledge about the NHIP. Factors such as gender (AOR: 1.80, 95% CI: 1.08–3.00), distance to the first point of contact (AOR: 2.15, 95% CI: 1.24–3.74), waiting time (AOR: 2.02, 95% CI: 1.20–3.42), availability of diagnostic services (AOR: 1.90, 95% CI: 1.05–3.45), availability of prescribed medicine (AOR: 3.90, 95% CI: 1.97–7.69), perceived service quality (AOR: 2.20, 95% CI: 1.15–4.20), and the behavior of service providers (AOR: 3.48, 95% CI: 1.04–11.63) were significantly associated with user satisfaction.

**Conclusion:**

The satisfaction level among NHIP users was deemed moderate. This study highlighted several factors, such as gender, distance to the first point of contact, waiting time, availability of diagnostic services and prescribed medicine, perceived service quality, and the behavior of service providers, as key determinants impacting user satisfaction. Recognizing the pivotal role of user satisfaction, health insurance stakeholders must prioritize it to ensure higher retention rates and coverage within NHIP.

## Introduction

According to the World Health Organization (WHO), universal health coverage (UHC) aims to ensure that everyone can access health services without suffering financial hardship [1]. However, the current situation is concerning, with an estimated 150 million people globally suffering from the financial catastrophe and 100 million people being pushed below the poverty line annually due to out-of-pocket (OOP) expenditure, particularly affecting low-income countries [2]. Due to these concerns, the shift from direct payments for healthcare to a prepayment social health protection mechanism has been considered a crucial step in mitigating the financial difficulties associated with healthcare expenses [3].

In the past, Nepal has launched various models of health insurance programs, ranging from programs within institutions like health facilities and cooperatives to government-funded community-based initiatives to achieve universal coverage [4, 5]. However, the sustainability of community-based health insurance programs was a major challenge. Various pre-paid programs implemented in Nepal were often isolated, lacked financial sustainability, had limited population coverage, and suffered from low retention of members [4]. Nepal, as a low and middle-income country with a poor financing mechanism, heavily relies on household OOP payments for financing health expenditures, which account for 54.2% of current health expenditures [6]. As an initiative to reduce OOP payments and improve financial protection among the population, the Government of Nepal launched the national health insurance policy in 2014 [7]. In 2015, the Government of Nepal formed the Social Health Security Development Committee (SHSDC), a semi-autonomous agency that started implementing NHIP in 2016 [8, 9]. The NHIP commenced in phases, starting from three districts (Kailali, Baglung, and Ilam) and subsequently expanded to cover all districts of Nepal by 2023 [10].

Under NHIP, a five-member family has to pay a contribution amount of Nepalese Rupees (NPR) 3,500 (around 26 USD) with an additional amount of NPR 700 (about 5 USD) for each additional member. Notably, the government provides full subsidy in the contribution amount for the elderly above 70 years, family members of ultra-poor households, people living with HIV, drug-resistant tuberculosis and leprosy, and those with complete disability. The benefit package with a ceiling of NPR 100,000 (around 763 USD) is provided for a five-member family and an additional NPR 20,000 (about 152 USD) is provided for each additional member. The NHIP provides coverage for emergency services, outpatient consultations, inpatient services, selected drugs, and diagnostic services [11].

The Health Insurance Act 2017 laid the foundation for an autonomous entity, the Health Insurance Board (HIB), entrusted with overseeing the NHIP and making enrollment mandatory for every citizen [12]. However, until 2023, enrollment is limited to the informal sector and is voluntary in practice, contributing to the challenge of low enrollment and high dropout rates currently faced by the NHIP [13].

User satisfaction is a multidimensional and broader concept that involves an individual’s perceptions, expectations, and experiences [14]. It is one of the barometers that shows the quality of a healthcare system [15, 16]. Improving service quality is equally important, along with the expansion of insurance coverage for the sustainability of the insurance program [17]. Therefore, it is necessary for service providers and insurance agencies to systematically measure user satisfaction to gauge the effectiveness of their services. While large bodies of literature from Sub-Saharan countries have documented the experiences of enrollment, their perception and satisfaction at the household level [18–23], studies exploring factors influencing household satisfaction in health insurance programs in Asia remain limited [24, 25]. Given Nepal’s unique socio-economic and health system context, coupled with its family-based health insurance modality, suggested that the factors established in other countries might also vary in the Nepalese context. Nonetheless, the current evidence base for the health insurance program in Nepal remains weak [26]. There have been some studies related to NHIP, mostly focused on determinants of enrollment, dropout rates, and service utilization [8, 27–31]. However, user satisfaction, a pivotal factor impacting NHIP renewal and fostering trust in the health system, has not been a primary focus in these studies. This study is expected to bridge the knowledge gap by generating evidence on factors influencing user satisfaction among NHIP members. The insights derived from this study could prove valuable to health insurance stakeholders in Nepal and other countries with similar settings for improving the modality of the health insurance system.

## Materials and methods

### Study design

The study employed a quantitative household-based cross-sectional study design. Since, the NHIP is household-based and the enrollment of other family members is linked to the identification of the household head, we selected the household head as a study participant.

### Setting for the study

Ilam district was chosen for the study because it is one of the three districts (Kailali, Ilam and Baglung) where NHIP was rolled out in the first phase [8]. Situated in eastern Nepal, Ilam is a hilly district with a population of 279,534, forming one of the fourteen districts within Koshi Province. The district comprises four urban municipalities and six rural municipalities. Ilam urban municipality and Maijogmai rural municipality were selected as study sites.

### Sample size

The Cochran 1963 formula was used to calculate the sample size [32].


n=z2p(1−p)d2

Where p = the level of household satisfaction with health insurance, which was taken as 55%, based on a previous study by Mitiku Kebede K et al. [18].

q = 1-p

d = desired level of precision or error allowance = 10% of 55% = 0.055

z = area under the normal curve (from the statistical z table) associated with a 95% confidence interval, which is 1.96.

The formula provided a total sample size of 315 participants. After adding a 10% non-response rate, the required sample size was 347 participants.

### Sampling technique

The study employed a multistage random sampling strategy to select the study population. Initially, the Ilam district was chosen purposefully as a study area. To ensure a balanced representation, one rural municipality (Maijogmai) and one urban municipality (Ilam) were selected; Maijogmai was chosen purposively as it was the only rural municipality of the Ilam district that had a facility i.e., Primary Health Care Center (PHCC) contracted with NHIP. Ilam municipality, on the other hand, was selected randomly through a lottery method.

**Fig 1:**
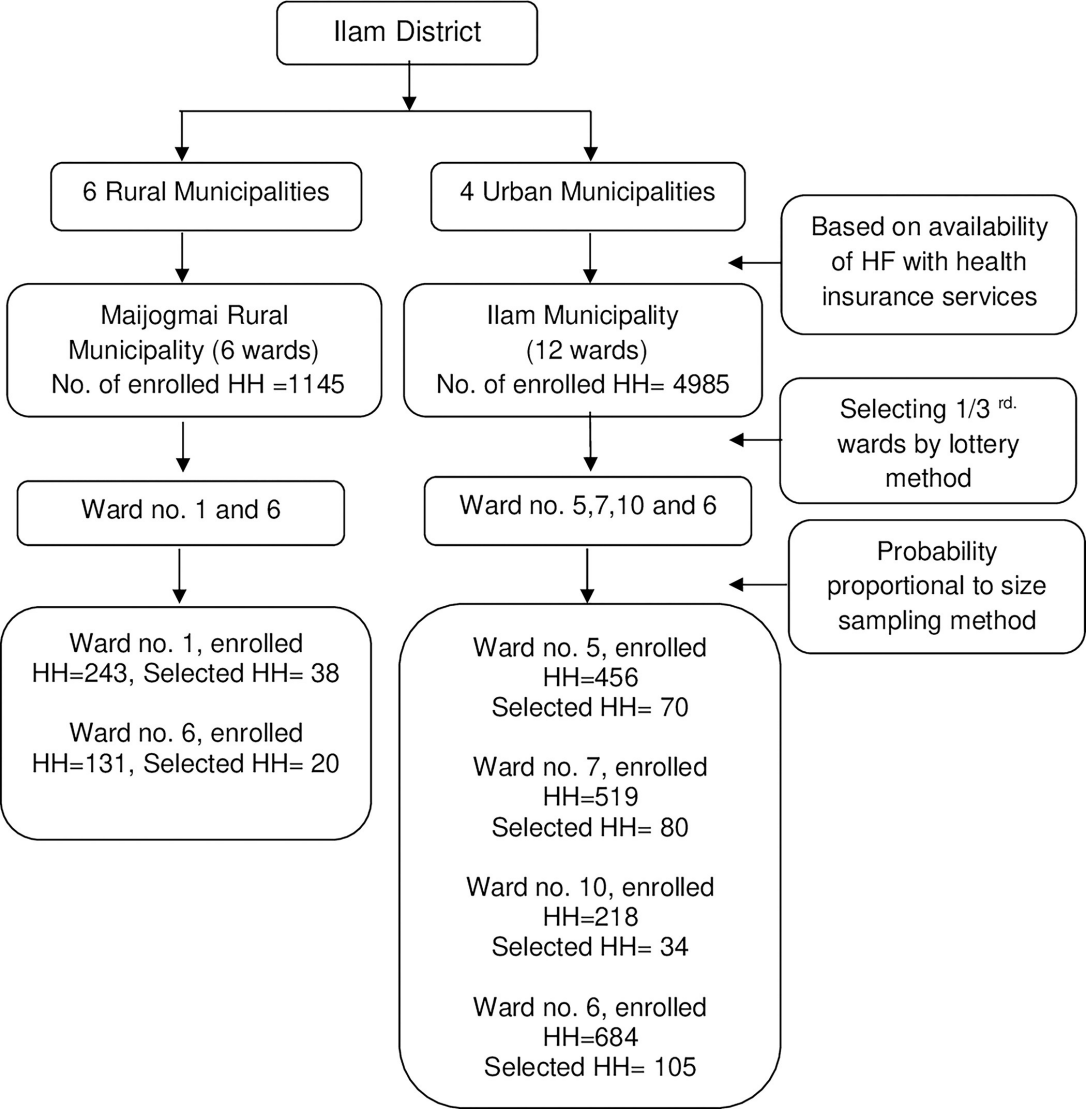
Sampling strategy.

Further, one-third of the wards were selected from each municipality using a lottery method i.e., two out of six wards for Maijogmai and four out of twelve for Ilam municipality. Based on the number of households enrolled in NHIP, the sample size was then allocated to each selected ward using probability proportional to size (PPS) sampling method. In Maijogmai, 38 out of 243 households were selected from Ward No. 1, and 20 out of 131 households were selected from Ward No. 6. Similarly, in Ilam municipality, 70 out of 456 households from Ward No. 5, 80 out of 519 households from Ward No. 7, 34 out of 218 households from Ward No. 10, and 105 out of 684 households from Ward No. 6 were selected (**Fig 1**).

For data collection, the HIB office of Ilam district provided a list of households with at least one year of enrollment in NHIP. A systematic random sampling method was employed to select households from the sampling frame. The sampling interval was determined by dividing the total number of households enrolled by the number of households to be sampled. Household selection was carried out with a fixed periodic interval of 6 after a random starting point. The enrolment assistants, volunteers responsible for enrolling families to the NHIP, helped in locating the sampled households. Thereafter, household heads meeting the inclusion criteria were interviewed for the study.

### Inclusion criteria for the study

Households with at least one year of enrollment in NHIP and those who had utilized its service at least once after being enrolled were selected as participants in the study.

### Data collection

Data was collected by the authors (RS and AS) using a pretested semi-structured questionnaire. It was developed by reviewing literature and modified to suit the Nepalese context [18, 22, 33, 34]. The questionnaire was initially developed in the English language, translated into the Nepali language and then back-translated into the English language. It was pre-tested among 35 households in the Suryodaya Municipality of Ilam district. The internal consistency of 14-item satisfaction questions was ascertained by calculating Cronbach’s alpha. The alpha value of 0.787 was obtained, which is acceptable. The data was collected from July to August 2022, through face-to-face interviews with the household heads. In cases where the household head was unavailable during the visit, the next available family member was interviewed.

### Study variables

The dependent variable was user satisfaction with NHIP, while the independent variables were socio-demographic and NHIP related variables (**Table 1**).

**Table 1:** Study variables.

S.N.	Variables	Categories of variables
**A.**	**Dependent variable**	
1.	User satisfaction with NHIP	Satisfied and dissatisfied
**B.**	**Independent variables**	
**a.**	**Socio-demographic variables**	
1.	Gender	Male and female
2.	Age	Less than 40 years, 40–59 years and 60 years or older
3.	Ethnicity	Brahmin/ Chhetri, Janajati, Dalit and Madhesi
4.	Education	Illiterate, literate (no formal education), basic level (class 1–8), secondary level (class 9–12) and others
5.	Marital Status	Married, widow/widower and single
6.	Family Type	Nuclear and joint
7.	Household size	< 5 and ≥ 5
8.	Presence of under 5 children	Yes and no
9.	Presence of elderly above 60 years	Yes and no
10.	Family’s main source of income	Agriculture and business/ service
11.	Working member of the family	1, 2 and ≥ 3
**b.**	**NHIP related variables**	
1.	Knowledge on NHIP	Poor, moderate and good
2.	Length of enrollment	< 4 years and ≥ 4 years
3.	First point of contact	Hospital and Primary Health Care Center
4.	Distance to First Point of Contact	< 30 min and ≥ 30 min
5.	No of times service used in the last one year	< 5 times and ≥ 5 times
6.	Types of visits	Outpatient service, inpatient service and emergency service
7.	Types of service used	Drugs and lab/diagnostic services
8.	Waiting time	< 60 min and ≥ 60 min
9.	Availability of diagnostic services	Frequently available and not frequently available
10.	Availability of prescribed medicines	Frequently available and not frequently available
11.	Perceived quality of service	Good and poor
12.	Behavior of service providers	Friendly, neutral and unfriendly
13.	Referral mechanism	Easy and lengthy process
13.	Affordability of premium	Affordable and difficult to afford

### Operational definition

#### User satisfaction with NHIP

It was measured using 14-item questions related to satisfaction i.e., insurance program-related factors (the amount of premium, time taken for the activation of the insurance card, schedule for paying the premium, information provided by the enrollment assistant, service coverage of insurance, behavior of insurance staff) and health service-related factors (availability of prescribed drugs, cleanliness of the health facility, adequacy of service providers, time given by the service provider, adequacy of medical equipment and rooms, behavior of service providers, perceived quality of care, waiting time at the health facility). It also included one extra question regarding overall self-rated satisfaction. All the responses were assessed on a five-point Likert scale from "very dissatisfied" to "very satisfied", with a score ranging from 1 to 5. All the scores of fourteen questions were added to compute the comprehensive satisfaction score. A receiver operating characteristic (ROC) curve was constructed to determine the cutoff value by using the comprehensive satisfaction score and self-rated overall satisfaction score. Self-rated overall satisfaction i.e., the leading question was re-categorized. For this, very satisfied and satisfied were merged as "satisfied" and the other three categories (neutral to very dissatisfied) were classified as "dissatisfied". As the aim of the study was to identify those who were fully satisfied with NHIP, those who stated neutral were considered dissatisfied [34]. Users with an overall comprehensive score equal to or above the cutoff value were categorized as satisfied, remaining others as dissatisfied.

#### Knowledge about NHIP

The respondent’s knowledge of NHIP was assessed using 14-item knowledge questions about the annual premium, annual benefit amount, membership renewal time and services under NHIP. A score of one was assigned to each correct answer. The total score obtained by the respondent was divided into three categories: poor knowledge (<50% score), moderate knowledge (50%-75% score), and good knowledge (>75% score) to obtain an overall knowledge level [27].

### Data management and analysis

The collected data were entered in Epi-Data version 3.1 and it was assessed daily for completeness and consistency of the information. The coded data was exported and analyzed in SPSS version 25. Descriptive analysis was done by calculating frequency and percentages for categorical variables, whereas mean and standard deviation were calculated for continuous variables. Binary logistic regression was used to estimate the association between independent variables and the binary outcome variable. Multivariable logistic regression analysis was used to determine the net effect of independent variables on the odds of user satisfaction level. The confidence level was set to 95% (p-value <0.05). All variables significant at a 20% significance level in bivariate analysis and expected cell count of more than five were taken for multivariable logistic regression analysis.

Prior to the inclusion of variables in the regression model for multivariate analysis, the variance inflation factor (VIF) was checked using collinearity diagnostics. None of the variables were found to be multicollinear, with the highest VIF value being 1.448. The regression model’s goodness of fit was determined using the Hosmer and Lemeshow chi-square test. The model was found to be a good fit with P> 0.05.

### Ethical approval and consent to participate

Ethical clearance was obtained from the Institutional Review Committee (IRC) of the B.P. Koirala Institute of Health Sciences, Dharan (Ref. no. 218/078/079). The purpose of the study was clearly explained to the respondents before data collection. Written informed consent was taken from the literate participants, while thumbprints were taken from the illiterate participants after reading out the consent form before the interview. Confidentiality of the information was maintained by limiting access to the data only to the authors. Voluntary participation and freedom to refuse by the participants at any stage were ensured by explaining the rights of the participants beforehand.

## Results

### Socio-demographic characteristics of the respondent

A total of 347 household heads participated in this study, making a response rate of 100%. **Table 2** describes the socio-demographic characteristics of the study population. Out of 347 participants, 51% were male. The participants’ ages ranged from 20 to 88 years, with a mean age of 45.7 years (SD = 13.9). Nearly half (47.8%) of the respondents belonged to the ethnic group Brahmin or Chhetri. Most of the respondents (43.2%) had completed education up to the secondary level, while nearly 5% were illiterate. Majority of the respondents were married (86.7%) and mostly had nuclear families (58.5%). More than half of the households (53.3%) had five or more members. About 20% of households had children less than five years of age and 41.8% had elderly family members (above sixty years). Most of the households (72.3%) were involved in business or service. Over half of the households (52.4%) had two working members within the family.

**Table 2:** Socio-demographic characteristics of the respondents (N = 347).

Characteristics	Number (N)	Percentage (%)
Gender		
Male	177	51.0
Female	170	49.0
Age	Mean age ± SD = 45.7± 13.9	
Less than 40 years	130	37.5
40–59 years	157	45.2
60 years or older	60	17.3
Ethnicity		
Brahmin/ Chhetri	166	47.8
Janajati	126	36.3
Dalit	51	14.7
Madhesi	4	1.2
Education		
Illiterate	17	4.9
Literate (no formal education)	50	14.5
Basic level (class 1–8)	73	21.0
Secondary level (class 9–12)	150	43.2
Others	57	16.4
Marital Status		
Married	301	86.7
Widow/widower	25	7.2
Single	21	6.1
Family Type		
Nuclear	203	58.5
Joint	144	41.5
Household size		
< 5	162	46.7
≥ 5	185	53.3
Presence of under 5 children		
No	276	79.5
Yes	71	20.5
Presence of elderly above 60 years		
No	202	58.2
Yes	145	41.8
Family’s main source of income		
Agriculture	96	27.7
Business/ Service	251	72.3
Working member		
1	103	29.7
2	182	52.4
≥ 3	62	17.9

### NHIP related factors

Only 31.1% of respondents had good knowledge about NHIP. Fifty-nine percent of the households had completed four or more years of NHIP membership. One in two households (51.6%) had the first point of contact (FPC) within a thirty-minute distance. In the last year, most visits were primarily for outpatient services (95.9%) and drugs (95.8%). The mean waiting time to visit a service provider in a health facility was 50 minutes (SD = 51.6). Around 63% and 70% of the study participants perceived that diagnostic services and prescribed medicines were not frequently available in the health facilities, respectively. However, the majority of the respondents (77.5%) expressed a positive perception regarding the quality of medicines provided under NHIP. The behavior of service providers was perceived as friendly by 34.9% of the respondents. The referral process was easy for 82% of the respondents. Likewise, 95.7% of households found the current annual premium of NPR 3500 affordable. Additionally, the government subsidized the contribution amount for 5% of households (**Table 3**).

**Table 3:** NHIP related factors (N = 347).

Characteristics	Number (N)	Percentage (%)
Knowledge on NHIP		
Poor	12	3.5
Moderate	227	65.4
Good	108	31.1
Length of enrollment	Mean ± SD = 3.82 ± 1.53	
< 4 years	142	40.9
≥ 4 years	205	59.1
First point of contact		
Hospital	278	80.1
PHCC	69	19.9
Distance to the first point of contact	Mean ± SD = 43.3 ± 43.1	
< 30 min	179	51.6
≥ 30 min	168	48.4
No. of times service used	Median (IQR)	= 3 (6–1)
< 5 times	226	65.1
≥ 5 times	121	34.9
Types of visit*		
Outpatient service	325	95.9
Inpatient service	25	7.4
Emergency service	83	24.5
Types of services used*		
Drugs	316	95.8
Lab/diagnostic service	226	68.5
Waiting time	Mean ± SD =	50.0 ± 51.6
< 60 min	196	56.5
≥ 60 min	151	43.5
Availability of diagnostic services		
Frequently available	130	37.5
Not frequently available	217	62.5
Availability of prescribed medicine		
Frequently available	107	30.8
Not frequently available	240	69.2
Perceived quality of service		
Good	269	77.5
Poor	78	22.5
Behavior of service providers		
Friendly	121	34.9
Neutral	203	58.5
Unfriendly	23	6.6
Referral mechanism (N = 183)		
Easy	150	82.0
Lengthy process	33	18.0
Affordability of premium		
Affordable	332	95.7
Difficult to afford	15	4.3

*Multiple choices, IQR = interquartile range

### Comprehensive satisfaction level

**Fig 2:**
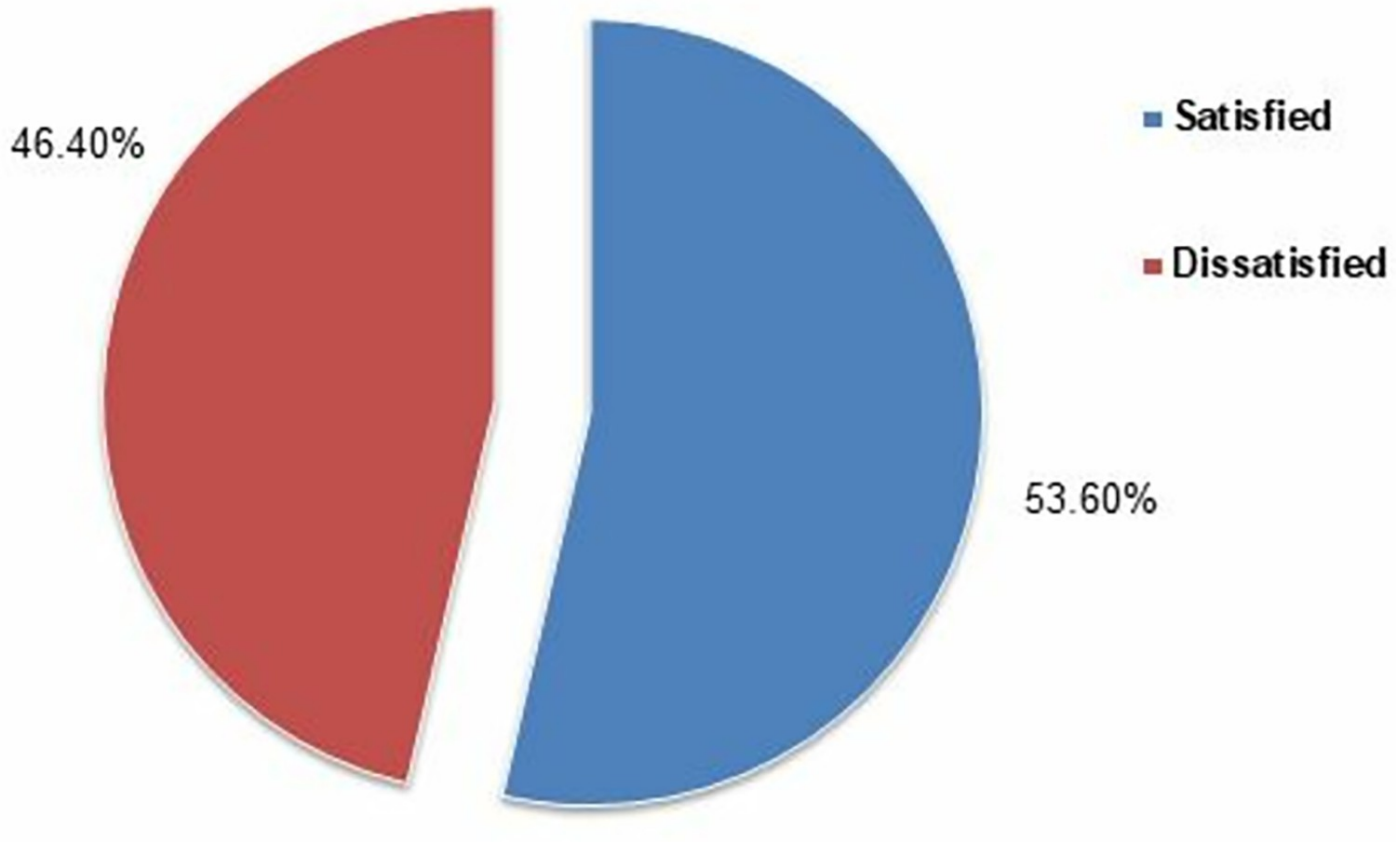
Comprehensive satisfaction level.

Overall, 53.6% were satisfied with the NHIP. The ROC curve gave a cutoff value of 44.5, where sensitivity was 0.710 and 1-specificity was 0.318, with the area under the curve being 0.738. A comprehensive satisfaction score greater than or equal to 44.5 was categorized as “satisfied” and all scores below 44.5 were categorized as “dissatisfied” (**Fig 2**).

### Factors associated with NHIP user satisfaction

The multivariate analysis (**Table 4**) shows that gender, distance to FPC, waiting time, availability of diagnostic service, availability of prescribed medicine, perceived quality of service and behavior of service providers were significantly associated with the NHIP’s user satisfaction.

**Table 4:** Factors associated with NHIP’s user satisfaction.

Explanatory Variables	Satisfied	Dissatisfied	AOR (95% CI)
	N (%)	N (%)	
Age			
less than 40 years	60 (32.3)	70 (43.5)	1
40–59 years	89 (47.8)	68 (42.2)	1.91(0.91–4.00)
60 years or older	37 (19.9)	23 (14.3)	1.58 (0.90–2.76)
Gender			
Male	105 (56.5)	72 (44.7)	1.80 (1.08–3.00)*
Female	81 (43.5)	89 (55.3)	1
Ethnicity			
Brahmin/Chhetri	104 (55.9)	62 (38.5)	1.85 (0.87–3.93)
Janajati	56 (30.1)	70 (43.5)	1.16 (0.54–2.49)
Others (Dalit, Madhesi)	26 (14.0)	29 (18.0)	1
Family’s main source of income			
Agriculture	59 (31.7)	37 (23.0)	1.06 (0.58–1.91)
Business/ Service	127(68.3)	124(77.0)	1
First point of contact			
PHCC	44 (23.7)	25 (15.5)	0.94 (0.46–1.88)
Hospital	142(76.3)	136 (84.5)	1
Distance to the first point of contact			
< 30 min	111(59.7)	68 (42.2)	2.15 (1.24–3.74)*
≥ 30 min	75 (40.3)	93 (57.8)	1
Waiting time			
< 60 min	120 (64.5)	76 (47.2)	2.02 (1.20–3.42)*
≥ 60 min	66 (35.5)	85 (52.8)	1
Availability of diagnostic services			
Frequently available	96 (51.6)	34 (21.1)	1.90 (1.05–3.45)*
Not frequently available	90 (48.4)	127 (78.9)	1
Availability of prescribed medicine			
Frequently available	86 (46.2)	21 (13.0)	3.90 (1.97–7.69)**
Not frequently available	100 (53.8)	140 (87.0)	1
Perceived quality of service			
Good	164 (88.2)	105 (65.2)	2.20 (1.15–4.20)*
Poor	22 (11.8)	56 (34.8)	1
Behavior of service providers			
Friendly	74 (39.8)	47 (29.2)	3.48 (1.04–11.63)*
Neutral	106 (57.0)	97 (60.2)	2.26 (0.69–7.34)
Unfriendly	6 (3.2)	17 (10.6)	1

* P < 0.05

** P< 0.001, AOR = Adjusted Odds Ratio, CI = Confidence Interval

Males had 1.8 times higher odds of being satisfied with NHIP compared to females (AOR: 1.80, 95% CI: 1.08–3.00). Households with a distance to FPC of less than 30 minutes had two times higher odds of being satisfied with NHIP compared to those with a distance of 30 minutes or more (AOR: 2.15, 95% CI: 1.24–3.74). Likewise, respondents who had to wait less than one hour to meet the service provider had two times higher odds of being satisfied with NHIP compared to those who had to wait one or more hours (AOR: 2.02, 95% CI: 1.20–3.42). Those who perceived diagnostic service as frequently available had nearly two times higher odds of being satisfied (AOR: 1.90, 95% CI: 1.05–3.45) compared to those who reported diagnostic service as frequently not available. Those who received the prescribed medicine regularly had four times higher odds of being satisfied with NHIP (AOR: 3.90, 95% CI: 1.97–7.69) than those who did not receive the prescribed medicines regularly, respectively. The respondents who perceived the quality of drugs under NHIP as good had two times higher odds of being satisfied compared to those who perceived it as poor (AOR: 2.20, CI: 1.15–4.20). Likewise, those respondents who found the behavior of service providers to be friendly had three times higher odds of being satisfied with NHIP than those who found it unfriendly (AOR: 3.48, CI: 1.04–11.63).

Age, ethnicity, family’s main source of income and FPC were not significantly associated with user satisfaction.

## Discussion

The study revealed a moderate level of satisfaction among users of Nepal’s NHIP, suggesting that all insured are not fully satisfied with the existing scheme of health insurance. Understanding factors affecting user satisfaction will help to reduce dropout in NHIP and thus improve financial protection for the population. A previous study also showed that, although the service utilization rate was high among the insured in the Ilam district, they were not fully satisfied with the service provided [35]. A similar finding was reported by a study in the Kaski district of Nepal, where user satisfaction was 52.5% [36]. User satisfaction with the health insurance scheme differs by country. Studies conducted in Ethiopia and Turkey showed user satisfaction of 54.7% and 53.3% respectively [18, 37]. In contrast to this, the level of satisfaction reported by a study in Nigeria is slightly lower (42.1%) [33] than in this study. However, user satisfaction with the Tanzanian Community Health Insurance Fund (70.5%) and Taiwan National Health Insurance (70%) was higher than in this study [38, 39]. User satisfaction appears closely tied to the quality of service, health insurance system and service coverage at a country level [24]; this might have resulted in variations in user satisfaction across different countries. Similarly, differences in tools and categorization used to measure satisfaction might have also affected the satisfaction level among the members of the insurance program.

Our study showed that males had higher odds of being satisfied with NHIP compared to females. Though females a have higher use of services under health insurance compared to males, as shown by a previous study conducted in the Ilam district [35], they have a higher level of dissatisfaction than males. Gender differences in user satisfaction need to be further explored. In contrast, studies conducted in Nigeria and Ethiopia have not shown any association between gender and user satisfaction [33, 40–42].

Travel time to reach FPC significantly affected user satisfaction in our study. It might be because people prefer health facilities within walking distance. Given the high transportation costs coupled with poor road infrastructure, spending a longer time to reach health facilities might have led to dissatisfaction among the insured. Studies have shown that people would prefer private health facilities with no insurance service despite their high cost rather than traveling longer distances [43–46]. Similar to this finding, a study conducted in Nigeria revealed physical accessibility to health facilities as an important factor leading to higher user satisfaction [47].

In this study, insured who had to wait longer in the health facility had a lower level of satisfaction. On average, they had to wait 54 minutes to see the doctor. It has emerged as one of the major areas of dissatisfaction with health services [31]. The possible reason might be that users may have higher opportunity costs related to the visit, i.e., this time could be used by them for other income-generating activities. This finding is in line with the results from countries like Ghana and Ethiopia [21, 40, 41, 48].

The availability of diagnostic services was significantly associated with user satisfaction. This finding is also supported by the studies conducted in Ethiopia [18, 19, 23]. Likewise, the availability of prescribed medicine showed a positive relationship with user satisfaction. A similar finding was shared by the studies conducted in Ethiopia, Iran and Uganda [23, 41, 49, 50]. In this study, diagnostic services and prescribed medicine covered by the benefit package were frequently available for only 37.5% and 30.8% of the users respectively, which depicts the condition of inadequate/dysfunctional equipment and poor stock management at the health facility level. Users who are frequently unable to get the services under NHIP are forced to pay OOP, leading to an increase in their level of dissatisfaction.

The study found that users who perceived the quality of service under NHIP to be good were more likely to be satisfied. This finding is supported by the studies conducted in Indonesia and China [51, 52]. This finding highlights the importance of improving the user experience at the health facility and enhancing the quality of the services. Though NHIP aims to provide quality services to the insuree, the quality of the services that HIB purchases is dependent on the services that the health facilities provide. Hence, health system strengthening is critical to improve service quality for the retention and satisfaction of users with NHIP.

The behavior of service providers was an important predictor of user satisfaction. Studies conducted in Bangladesh, Ghana and Ethiopia have shown that service providers’ friendliness led to an increase in user satisfaction [19, 22, 24]. The insured’s poor perception of service providers’ behavior can force them to seek care from other health facilities at their expense, which can result in their dropping out of health insurance [27, 53].

To our knowledge, this is one of the first few studies attempting to identify the factors associated with user satisfaction in NHIP in a community setting. However, it has some limitations. The cross-sectional study was conducted in one of the hilly districts of Nepal. Hence, the findings generated from this study can be generalized to similar settings only. Some of the explanatory variables used in the study were related to the perceptions of the insuree, which might have suffered from information bias. Along with this, respondents were asked to provide information about the service used in the last one-year period, which might have led to recall bias. Nevertheless, these findings highlight the urgent need to reduce distance to FPC by expanding service sites and improving service quality for a more satisfactory NHIP experience. Strengthening the health system alongside the insurance program is crucial to meet people’s NHIP expectations. It is also important to improve health insurance literacy at the population level so that people enroll and continue to join NHIP.

## Conclusion

We found a moderate level of user satisfaction with NHIP among the insured population of the Ilam district. Gender, distance to the FPC, waiting time, availability of diagnostic service, availability of prescribed medicine, perceived quality of service and behavior of the service providers were the predictors of user satisfaction. These findings highlight the multifaceted nature of factors influencing satisfaction levels, ranging from accessibility issues like distance and waiting times to the quality and availability of healthcare services. Based on these results, our study suggests that the Health Insurance Board, Ministry of Health and Population and government agencies at all levels should prioritize efforts to enhance the quality and availability of health services within HIB-contracted health facilities. Thus strategic focus is imperative for addressing the identified predictors of user satisfaction. Improvement in these areas not only has the potential to elevate satisfaction levels but also contribute to the overall effectiveness and success of the NHIP.

## Supporting information

S1 Data (SAV)
